# Moving on From Sipuleucel-T: New Dendritic Cell Vaccine Strategies for Prostate Cancer

**DOI:** 10.3389/fimmu.2021.641307

**Published:** 2021-03-29

**Authors:** Sarah I. M. Sutherland, Xinsheng Ju, L. G. Horvath, Georgina J. Clark

**Affiliations:** ^1^Dendritic Cell Research, ANZAC Research Institute, Concord, NSW, Australia; ^2^Faculty of Medicine and Health, University of Sydney, Sydney, NSW, Australia; ^3^Department of Medical Oncology, Concord Repatriation General Hospital, Concord, NSW, Australia; ^4^Department of Medical Oncology, Chris O'Brien Lifehouse, Camperdown, NSW, Australia; ^5^Garvan Institute of Medical Research, Darlinghurst, NSW, Australia

**Keywords:** dendritic cell, vaccine, prostate cancer, tumor, immune system, immunotherapy

## Abstract

Tumors evade the immune system though a myriad of mechanisms. Using checkpoint inhibitors to help reprime T cells to recognize tumor has had great success in malignancies including melanoma, lung, and renal cell carcinoma. Many tumors including prostate cancer are resistant to such treatment. However, Sipuleucel-T, a dendritic cell (DC) based immunotherapy, improved overall survival (OS) in prostate cancer. Despite this initial success, further DC vaccines have failed to progress and there has been limited uptake of Sipuleucel-T in the clinic. We know in prostate cancer (PCa) that both the adaptive and the innate arms of the immune system contribute to the immunosuppressive environment. This is at least in part due to dysfunction of DC that play a crucial role in the initiation of an immune response. We also know that there is a paucity of DC in PCa, and that those there are immature, creating a tolerogenic environment. These attributes make PCa a good candidate for a DC based immunotherapy. Ultimately, the knowledge gained by much research into antigen processing and presentation needs to translate from bench to bedside. In this review we will analyze why newer vaccine strategies using monocyte derived DC (MoDC) have failed to deliver clinical benefit, particularly in PCa, and highlight the emerging antigen loading and presentation technologies such as nanoparticles, antibody-antigen conjugates and virus co-delivery systems that can be used to improve efficacy. Lastly, we will assess combination strategies that can help overcome the immunosuppressive microenvironment of PCa.

## Introduction

Immune evasion has long been recognized as a problem in prostate cancer (PCa). To date checkpoint inhibitors that aim to release the “brakes” on T cell expansion have proved disappointing (1–3). Dendritic cells (DC) bridge the gap between the innate and adaptive immune response, playing a crucial role in tipping the direction toward inflammation or tolerance. Manipulating this balance through DC vaccine therapy has therapeutic potential. This is not a novel concept (4); in 2010, Sipuleucel-T was the first DC therapy approved by the FDA for the treatment of metastatic castrate resistant prostate cancer (mCRPCa) (5). Our understanding of DC biology has vastly increased over the last decade, yet no further DC therapy has been FDA approved. In this review we will assess the strengths and weaknesses of prior approaches and then look at the potential of new technologies to drive improvements. Here we review how these technologies apply to PCa and suggest combination therapies that might overcome the immunosuppressive microenvironment leading to better clinical outcomes.

## Dendritic Cell Vaccination in PCa

In PCa DC are dysfunctional and key orchestrators of its immunosuppressive microenvironment (6–11). Sipuleucel-T demonstrates that taking antigen presenting cells (APC) from PCa patients, pulsing them with tumor peptide and inducing their maturation prior to returning them back to patients, primes T cells that track to the tumor itself (12). In a pooled analysis of two, phase III, randomized control trials (RCT) in minimally or asymptomatic mCRPCa, Sipuleucel-T, improved overall survival (OS) to 23 months from 19 months [Hazard Ratio (HR) 1.50, 95% CI: 1.10–2.05, *p* = 0.01] (13). This OS benefit was corroborated by a third trial where OS was similarly increased by 4.1 months (5). Despite such promise the use of Sipuleucel-T in the clinic remains low.

One reason is skepticism over the trial results. It has been proposed that the control arm did worse than anticipated and that the benefit seen was in fact due to the harm of apheresis, where fewer PBMC were returned to patients in the control arm than the treatment arm (14). This has not been helped by a plethora of further DC-based therapy trials performed with monocyte-derived DC (MoDC) that despite showing immunological responses, have failed to show real clinical benefit.

### MoDC Vaccination

**Figure 1:**
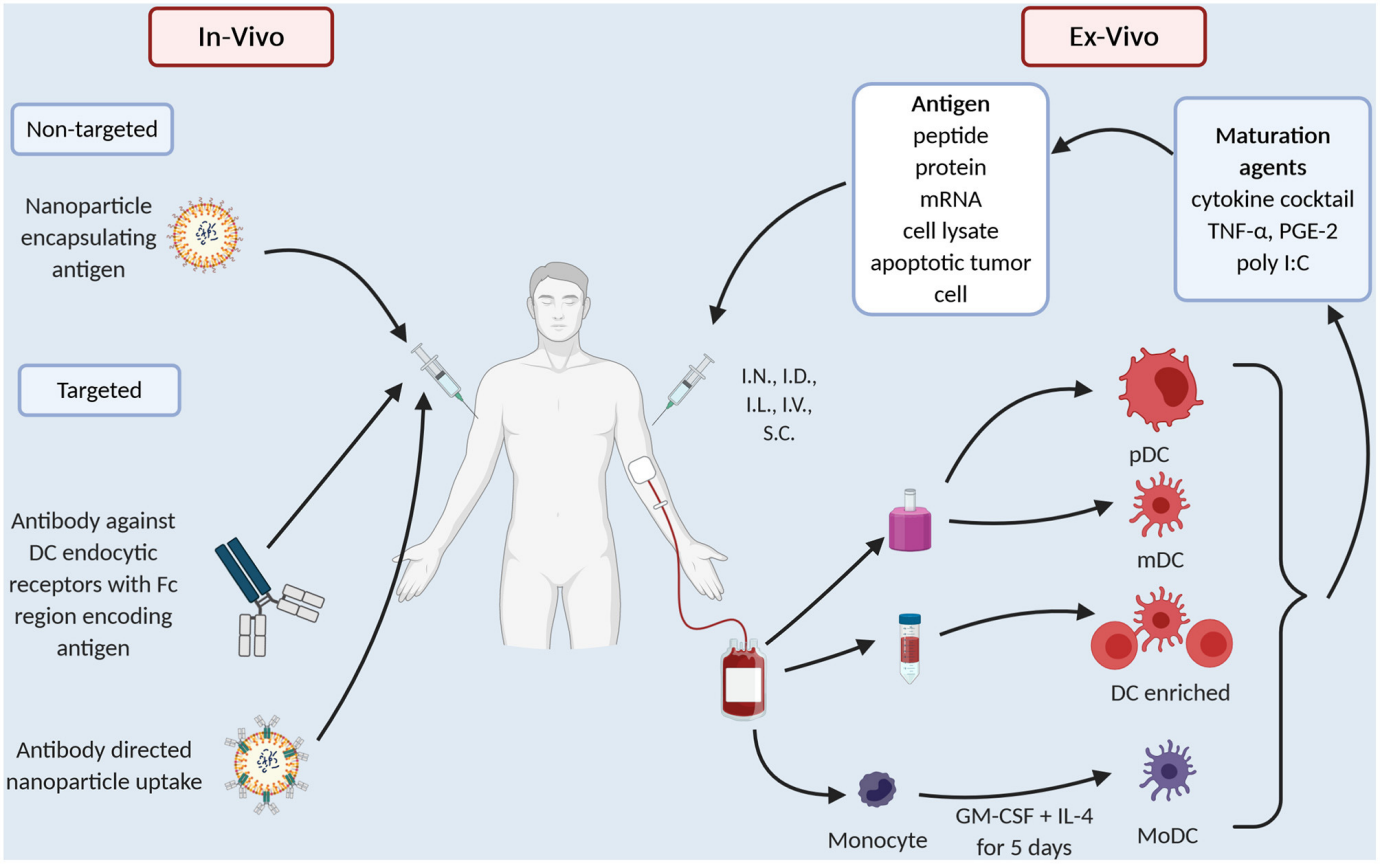
Current dendritic cell vaccination technologies.

The common technical issue in any DC preparation is the low prevalence of DC in the peripheral blood, ranging 0.1–1% of peripheral blood mononuclear cells (PBMC) (15). Thus, early DC preparations such as Sipuleucel-T use a density gradient to prepare an APC enriched preparation (Figure 1). “Second generation” DC vaccines use strategies that differentiate monocytes into dendritic like cells called MoDC (Figure 1), creating a more readily available source of APC as monocytes make up ~10% of PBMC compared to 1% for DC. MoDC are prepared by separating CD14^+^ cells from PBMC either by their ability to adhere to plastic overnight culture or by anti-CD14 microbeads and magnetic separation. CD14^+^ cells are then cultured with cytokines, typically GM-CSF and IL-4 for 4–5 days, after which they display an immature DC like phenotype (16). When cultured with tumor antigen in the form of peptide or protein these cells cross-present and induce T cell proliferation (16). In melanoma patients, whilst only a small proportion i.e., 4% of I.D., injected DC, migrate to local lymph nodes but those that do activate CD8^+^ T cells in a melanoma model, thus overcoming microenvironment of melanoma (17). There have been several clinic trials in PCa with MoDC (Table 1). They vary in their mode of antigen delivery (protein, peptide, apoptotic tumor cells, cell lysate from tumor cell lines or mRNA) (Table 1, Figure 1), whether the MoDC are immature or mature and if matured what activation agent was used (Table 1, Figure 1). All these nuances have a profound impact on efficacy and applicability thus it is worth exploring these differences in more detail.

**Table 1:** Published DC vaccination trials in prostate cancer.

**Cell Type and Maturation**	**Antigen**	**Trials**	**Population**	**Phase**	**Pt #**	**Intervention:**	**Route ± adjuvant**	**Outcome**
**Immature MoDC**						**Peptide (HLA-A2)**	
	PSMA-P1 and PSMA-P2	Murphy et al. (18)	CRPCa	1	51	Arm 1 + 2: Peptide (*n* = 20) Arm 3: DC (*n* = 12) Arm 4 + 5: DC Vaccine (*n* = 19) Arm 1 + 4: PSMA-1 Arm 2 + 5: PSMA-2	I.V.	**Primary:** *Safety:* (hypotension 24/51, fatigue 3/51 **Secondary:** *Immunological:* T cell proliferation in response to peptide (↑ in HLA-A2+ DC vac pts) *Clinical:* Peptide (PR 2/19, SD 2/19) DC (PR 0/12, SD 2/12) DC Vaccine (PR 5/20, SD 3/20)
	PSMA-P1 and PSMA-P2	Murphy et al. (19)	CRPCa	II	33		I.V.	*Clinical:* CR 2/25, PR 6/25, 1/25 SD
	PSMA-P1 and PSMA-P2	Murphy et al. (20)	Recurrent CSPCa	II	41		I.V.	*Clinical:* CR 1/37, PR 10/37
	PSMA-P1 and P2 + KLH	Murphy et al. (21)	CRPCA	II	17		I.V.	*Safety:* fever, fatigue, muscle cramps *Clinical:* CR 1/17, PR 3/17
	PSMA_4−12_	Knight et al. (22)	CRPCa—HLA-A2 positive	I	12	Cells irradiated prior to infusion	S.C.	*Safety:* Fatigue 4/12, fever 4/12, pain 4/12 *Immunological:* ELISPOT 0/12
	PSA_146−154_	Perambakam et al. (23)	CSPCa	I	28	Cohort 1: high risk locally advanced disease Cohort 2: metastatic disease Arm A: Peptide + GM-CSF (I.D.) Arm B: MoDC	I.L.	*Immunological:* DTH Arm A 9/14 Arm B: 5/14
						**Protein**		
	PSA	Barrou et al. (24)	bcrCSPCa	II	26	Used GM-CSF and IL-13 rather than IL-14	I.V., S.C., I.D.	*Safety:* 3/24 macular rash, 2/24 G2 increase in bilirubin, 1/24 asthenia, 1/24 halitosis *Clinical:* Circulating tumor cells 6/6. PSA response 0/24. *Immunological:* ELISPOT response to PSA 4/24 developed a response on treatment No antibody response.
						**Cell-lysate**	
	LNCaP, DU145	Pandha et al. (25)	CRPCa	I/II	11		I.D.	**Primary:** *Feasibility:* Vaccine produced in 11/11 pts *Safety:* nil significant local or systemic toxicity **Secondary:** *Clinical:* PSA response 0/11, ↑PSADT in 3/11 *Radiological:* CR 0/11, PR 0/11, SD 4/11 *Immunological:* DTH response 0/11, ELISPOT response 6/11
						**mRNA**	
	PSA	Heiser et al. (26)	mPrCa	I	16		I.V., I.D.	*Feasibility:* assigned cell dose given 12/13 patients *Safety:* fever and flu-like sx 4/13, injection site reaction 4/13 *Immunological Response:* ELISPOT 9/9
**Mature MoDC**						**Peptide (HLA-A2)**	
TNF-α, PGE-2	PSCA_14−22_ PSA1_141−150_ PSA2_146−154_ PSA3_154−163_	Thomas-Kaskel et al. (27)	CRPCa HLA-A2+	I/II	12	Arm 1: PSCA peptide + PSA peptides Arm 2: cell penetrating peptide (CPP)-PSCA + PSA peptides	S.C.	**Primary:** *Feasibility:* 10/12 pts received at least 3 vaccinations *Safety:* no reported toxicity **Secondary:** *Immunological:* DTH to tumor peptide 5/10, Tetramer 1/10 *Clinical:* SD 4/10
	PSA1_141−150_ PSA2_146−154_ PSA3_154−163_	Hildenbrand et al. (28)	CRPCa	I	15		I.D.	**Primary:** *Clinical Response:* PR 1/12, SD 4/12 *Biochemical Response:* 1/12, ↑PSADT **Secondary:** *QOL:* no change *Immunological:* DTH response: 9/12 *Feasibility:* 12/15 enrolled evaluable *Safety:* fever 11/12, local erythema 11/12, 6/12 bone pain, 3/12 slight articular pain, 1/12 insomnia
	PSA_146−154_ PSMA_4−12_ PAP_299−307_	Zhuang et al. (29)	CRPCa	I	16		S.C.	*Immunological:* DTH response 4/12 *Clinical:* PR 3/16, 7/16 SD
Poly I:C	PSMA_154−163_ Survivin_95−104_	Xi et al. (30)	Non-mCRPCa	II	21	Arm 1: DC vaccine (*n* = 11) Arm 2: Docetaxel and prednisone (*n* = 11)	S.C.	*Safety:* local reaction 4/11, *Immunological:* DTH response 11/11, ELISPOT increased compared to docetaxel arm (*p* = 0.048) *Clinical:* DC arm vs. docetaxel *PR:* 3/11 vs. 0/11, SD 6/11 vs. 5/11
Cytokine cocktail	PSA3_154−163_ PSMA_4−12_ Prostein_31−39_ Survivin_950104_ Trp-p8_187−195_	Fuessel et al. (31)	CRPCa	I	8		I.V., I.D.	*Safety:* local reaction *Clinical:* PSA response 1/8 *Immunological:* ELISPOT 4/8
	PSCA_14−22_ PAP_299−307_ PSMA_4−12_ PSA_154−163_	Waeckerle-Men et al. (32)	mCRPCa	I	6		I.D.	*Safety:* local reaction 5/6 *Clinical:* ↑PSADT 3/6 *Immunological:* ELISPOT 3/6, DTH 3/6
						**Protein**		
Cytokine cocktail	Tn-MUC1 + KLH	Scheid et al. (33) NCT00852007	Non-mCRPCa	I/II	17	Tn-MUC1+	I.N., I.D.	*Safety:* local reaction 16/17, G1 fatigue 1/, G1 insomnia *Clinical Response:* biochemical 0/16, PSADT increased in 11/16. *Immunological:* Intracellular response in CD4^+^ CD8 T cells in 2/16, CD4 in 1/16, CD8 in 2/16.
						**mRNA**		
Cytokine cocktail	mRNA from DU145, LNCaP, PC3	Mu et al. (34)	CRPCa	I/II	20	Arm A: I.N. (*n* = 10) Arm B: I.D. (*n* = 9)	I.N. or I.D.	*Safety:* injection site reactions *Immune response:* ELISPOT 10.19 *Clinical:* Reduced PSA slope 13/19
						**Cell lysate**		
Cytokine cocktail	DU145 LNCaP PC3	Reyes et al. (35)	CRPCa	I	20		S.C.	*Safety:* 8/20 local erythema and pain, 1/20 hypertension *Feasibility:* 14/20 completed study protocol *QOL:* no change *Immunological:* ELISPOT 7/14, DTH 9/14. *Clinical:* PSA response 6/14
						**Apoptotic cell line**		
TNF-α, PGE-2	LNCaP	Frank et al. (36) NCT00289341	bcrCSPCa or CRPCa	1	24	Arm1 : Vaccine weeks 1–7 (*n* = 12) Arm 2: Placebo weeks 1–7, vaccine weeks 8–14 (*n* = 12)	S.C	*Safety:* injection site reactions in the first 7 weeks, 11/12 in arm 1 vs. 2/12 arm 2 *Immunological:* DTH response 16/24, T cell proliferation response *Clinical:* ↑PSADT prevaccine vs. post vaccine (*P* = 0.003)
						**Transfected DC**	
	PSMA protein	Sonpavde et al. (37)	mCRPCa	I	18	MoDC transfected with adenoviral vector Ad5f35 encoding inducible human CD40 injected I.D. then given rimiducid IV 24 h later to induce CD40 expression on DC	I.D.	*Safety*: 18/18 local reaction, fatigue 6/18, myalgias 5/18, anemia 4/18, diarrhea 4/18, respiratory tract infection 4/18, hypocalcaemia 4/18, arthralgia 4/18 *Clinical*: PSA response 1/18, Radiological: 2/18
**Enriched DC prep**						**Protein**		
	PA2024 (GM-CSF and PAP)	Burch et al. (38)	CRPCa	I	13	Two infusions of DC with PAP alone and then three infusions of PA2024	I.V.	*Safety:* G1-2 fever 5/13, G1-2 myalgia 5/13, G1-2 fatigue 6/13, G3 fatigue 1/13, local reactions 4/13 *Immunological:* T cell proliferation 9/9, *Clinical:* PSA response 3/12
	PA2024 (GM-CSF and PAP)	Small et al. (39)	CRPCa	I/II	31	Arm 1: Sipuleucel-T Arm 2: Sipuleucel-T as well KLH loaded DC (*n* = 5)	I.V.	*Safety:* febrile reactions 15/102, G3 febrile reactions 2/102, myalgias 2/31, fatigue 1/31, urinary symptoms 5/31 *Immunological:* T cell proliferation 10/26, 16/31 Antibody response 16/31 *Clinical:* PSA response 3/31
	Mouse PAP	Fong et al. (40, 41)	PrCa	I	21	Arm 1: I.V. (*n* = 9) Arm 2: I.D. (*n* = 6) Arm 3: I.L. (*n* = 6)	I.V., I.L., I.D.	*Safety:* Transfusion reactions in 2/18 I.V. injections *Immunological:* T cell proliferation against mPAP 21/21 pts. Ag specific IFN-γ response 0/9 I.V., 4/6 I.D., 3/6 I.L.
	PA2024 (GM-CSF and PAP)	Fong et al. (12) NCT00715104	Localized PrCa	II	42	Three doses Neoadjuvant treated prior to planned RP Arm 1: 4th injection 12 weeks post RP Arm 2: NO boost		*Safety:* fatigue, oral paresthesia *Immunological:* 57% pts had a 3-fold increase in tumor interface T cells
	PA2024 (GM-CSF and PAP)	Higano et al. (13)	Asymptomatic CRPCA	III	147	Arm 1: Placebo Arm 2: Sipuleucel-T	I.V.	*Clinical:* OS 19 vs. 23.2 months (HR 1.5, CI 1.1–2.05, *p* = 0.011) TTP 10 vs. 11 months (HR 1.26 0.95–1.58, *p* = 0.111)
	PA2024 (GM-CSF and PAP)	Kantoff et al. (5)	Asymptomatic CRPCA	III	512	Arm 1: Sipuleucel-T Arm 2: Placebo	I.V.	*Clinical:* OS 25.8 vs. 21.7 months (HR 0.78 CI 0.61–0.98, *p* = 0.03)
	PA2024 (GM-CSF and PAP)	Beer et al. (42)	bcrCSPCa	III	176	Pts with biochemical recurrence after RP were given 3–4 months of ADT and then randomized to: Arm 1: Sipuleucel-T Arm 2: Placebo	I.V.	**Primary:** *Biochemical Failure PSA > 3.0:* 18 vs. 15.4 months HR 0.93, *p* = 0.73) **Secondary:** *PSADT:* ↑PSADT 48% (*p* = 0.038) OS
**DC**						**Peptide (HLA-A2)**	
CD1c	PSA_174−183_ PSMA_711−719_ PAP_299−311_ Control peptides: FMP GILGFVFTL KLH	Prue et al. (43)	Asymptomatic mCRPCa (HLA-A2)	I	14	All 3 injections of CD1c: Arm 1: I.D. 1 × 10^6^ Arm 2: I.D. 1–5 × 10^6^ Arm 3: I.V. 1 × 10^6^ Arm 4: I.V. 1–5 × 10^6^	I.V. or I.D.	**Primary:** *Safety:* fever and pain *Feasibility:* 12/12 underwent leukapheresis and vaccination, 11/12 received 2nd vaccination **Secondary:** *Immunological:* DTH response 0/12, ELISPOT response 0/12, Pentamer positive CD8^+^ T cells 0/12 *Clinical:* PSA response 0/12
CD1c pDC (protamine and mRNA)	NY-ESO-1_157−165_ NY-ESO-1 (peptivator) MAGE-C2_336−34_ MUC1 (peptivator) KLH (control)	Westdorp et al. (44) NCT02692976	Chemo naive CRPCa (HLA-A2)	II	21	Arm 1: mDC vaccination Arm 2: pDC vaccination Arm 3: mDC and pDC vaccinations	I.N.	*Safety:* anemia 15/21, flu like symptoms 10/21, fatigue 8/21 *Immune Response:* Dextramer positive T cells to NY-ESO-1 5/21, MAGE-C2 4/21, MUC-1 2/21 Antigen Specific CD8^+^ T cells in DTH Response: 15/21 pts—no difference between arms *Clinical Response:* PSA response 2/21, Radiological 1/21
						**Combination therapy**	
Mature MoDC (poly I:C)	Cell lysate (LNCaP)	Podrazil et al. (45)	CRPCa	I/II	25	7 days of metronomic cyclophosphamide then 2 doses of vaccine and then 3 weekly docetaxel and vaccine	S.C. with Imiquimod	*Safety:* fatigue 17/350 *Immunological:* Intracellular cytokine response to PSA 11/23, MAGE-A1 6/23, MAGE-A2 3/23 *Antibody Response:* PSA 6/23, Mage A3 8/23 *Clinical:* PSA response 9/23
Mature MoDC (cytokine cocktail)	mRNA PAP and PSA	Kongsted et al. (46) NCT01446731	CRPCa	II	43	Arm 1: Docetaxel 75 mg/m^2^ every 3 weeks Arm 2: Docetaxel 75 mg/m^2^ every 3 weeks DCvac twice every 3 weeks for cycles 1–4 then once cycles 5–10	I.D.	**Primary:** Development of measurable peripheral immune *Response:* ELISPOT: 9/18, DTH: 3/18 **Secondary:** *Safety and Toxicity:* local reactions and rash, *Discontinuation of Treatment:* 21.1 vs. 57% *PSA Response:* 58 vs. 38%, *p* = 0.21 *PFS:* 5.5 vs. 5.7 months (*p* = 0.62) *DSS:* 21.9 vs. 25.1 months (*p* = 0.60)
DC enriched	PA2024 (GM-CSF and PAP)	Twardowski et al. (47)	mCRPCa	II	51	Arm A: sipuleucel-T alone (*n* = 24) Arm B: RT to single metastatic site followed by sipuleucel-T (*n* = 25)	I.V.	**Primary:** *Safety:* G2 fatigue 1/24 vs. 3/25 **Secondary:** ELISPOT IFNy ↑in Arm A compared to B (*p* = 0.028). PFS 2.46 vs. 3.65 months (*p* = 0.06)
DC enriched	PA2024 (GM-CSF and PAP)	Antonarkis et al. (48) NCT01431391	bcrCSPCa	II		Arm A: Sipuleucel-T followed by ADT 2 weeks after Arm B: ADT for 12 weeks then Sipuleucel-T	I.V.	**Primary:** ELISPOT—approx. 2-fold higher for Arm A than Arm B (*p* = 0.001) **Secondary:** Time to PSA progression 21.8 vs. 22.6 (*p* = 0.357)
DC enriched	PA2024 (GM-CSF and PAP)	Scholz et al. (49)	mCRPCa NCT01832870	I	9	Ipilimumab and Sipuleucel-T	I.V.	*Safety:* well tolerated only 1 G1 rash *Immunological:* increase in humeral immunity against PA2024 and PAP
Poly I:C	Cell lysate (LNCaP)	Fucikova et al. (50) EudraCT 2009-017259-91	bcrCSPCa	I/II	27	1 week of metronomic cyclophosphamide then DC vaccine every 2–6 weeks for approx. up to all manufactured doses on average 12	S.C. with Imiquimod	*Immunological:* IFN-γ specific T cells to PSA 12/27, MAGE 6/27 *Antibody Response to:* PSA 9/27, MAGE 9/27 *Clinical:* increase in PSADT 22/25
DC enriched	PA2024 (GM-CSF and PAP)	Small et al. (51) NCT01487863	mCRPCA	II	69	Arm A: concurrent Sipuleucel-T and abiraterone Arm B: Sipuleucel-T for 10 weeks then abiraterone	I.V.	No difference in immune response
DC enriched	PA2024 (GM-CSF and PAP)	Rini et al. (52) NCT00027599	bcrCSPCa	I	22	Sipuleucel-T and bevacizumab	I.V.	*Clinical:* ↑PSADT 6.9 vs. 12.7 months post treatment (*p* = 0.01)
DC enriched	PA2024 (GM-CSF and PAP)	Jha et al. (53)	mCRPCa	II	46	Arm A: Sipuleucel-T + indoximod Arm B: Sipuleucel-T	I.V.	*Clinical:* PSA progression no diff PFS 10.3 vs. 4.1 months (*p* = 0.011)

*Cytokine cocktail (TNF-α, IL-1β, IL-6, PGE2), mCRPCa, metastatic castrate resistant prostate cancer; bcrCSPCa, biochemical recurrence of castrate sensitive prostate cancer; DTH, delayed hypersensitivity, antigen specific response reported; PSADT, PSA doubling time; I.V., intravenous; I.N., intranodal; I.D., intradermal; S.C., subcutaneous*.

### Mature vs. Immature DC

Firstly, early trials used immature MoDC and as one would expect, immature MoDC have reduced expression of activation markers, reduced ability to stimulate T cells (54, 55) and reduced ability to migrate (55). A meta-analysis that extracted individual patient data from 10 clinical trials of DC vaccines in PCa confirmed that immature MoDC preparations had less clinical benefit than mature MoDC (56). In melanoma patients immature and mature MoDC preps were compared head-to-head, again immature MoDC were less effective (57).

Different maturation agents have been used (Table 1, Figure 1) and at least *in vitro* they activate different gene expression profiles in the MoDC which in turn causes differing T cell responses (58). Broadly, maturation agents haven been chosen that are GMP grade, induce activation markers and produce MoDC that stimulate T cells toward a type 1 helper T cell response. Human cytokine cocktail, consisting of TNF-α, IL-1β, IL-6, PGE2, has been most frequently used in MoDC trials (Table 1). This mix produces mature MoDC with a superior ability to stimulate T cells than immature MoDC (59) and improved migratory capacity to mobilize DC to lymph nodes where they can prime T cells (60). However, there is data that these MoDC preferentially recruit T-regs, thus, potentially dampening any immune response initiated (61, 62).

Polyinosinic-polycytidylic acid [poly(I:C)] is a synthetic analog of dsRNA and is a clinical grade TLR3 agonist that matures DC (63). These DC, unlike those produced by the cytokine cocktail, produced high levels IL-12 (64) which directs a Th1 type T cell response. *In vitro* experiments suggest better antigen specific T cell proliferation and less T reg development (60, 62). In clinical trials Poly I:C matured MoDC vaccines are reportedly well-tolerated producing immunological and clinical responses (50). However, there are no clinical trials that compare Poly (I:C) matured MoDC with cytokine cocktail matured MoDC directly.

A third combination of CD40L with IFN-γ has shown promise, similarly increasing IL-12 cytokine production (65). Whilst this combination has not been used in PCa, CD40L has been used in other cancer vaccines. In resected metastatic colorectal cancer, a small, randomized phase I DC vaccine trial randomized tumor lysate pulsed MoDC cultured with or without recombinant CD40L. CD40L induced CD86 and CD83 expression on DC but in this small study of only 26 patients, CD40L did not improve anti-tumor specific T cell proliferation, IFNγ ELISPOT response, DTH response or relapse free survival (66). Similarly, in melanoma patients where CD40L was compared to cytokine cocktail, no difference was found in immunological response (67).

There are numerous reasons why we do not see clinical effect of different DC maturation strategies despite promising preclinical data. One is that small gains in maturation state *in vitro* maybe overpowered by the immune environment *in vivo*. One strategy that aims to control this is the use of viral vectors to genetically modify DC. In PCa Sonpavde et al. (37) showed feasibility, safety, and the development of a peripheral immune response when DC were transfected with inducible human CD40 (37). In this trial an adenovirus vector was used to transfect DC with human CD40 that had its cytoplasmic domain fused to ligand-binding domains and a membrane-targeting sequence to allow CD40 to be regulated by lipid-permeable dimerizing drugs, in this case rimiducid (68). This allows control over the timing of CD40 expression. DC vaccine was given and 24 h after injection, when DC have migrated to the lymph node and are in close contact with T cells, rimiducid is given to activate CD40. In this phase 1 study, 86% of patients had stable disease, with just 10% with a partial response (37). In PCa PSA kinetics reported as PSA doubling time (PSADT) are an indicator of prognosis with a shorter PSADT indicating a worse prognosis (69). In this study, 53% of patients had an increase in their PSADT, a surrogate marker for improved clinical outcome. This proof of concept shows that we can co-ordinate both timing and activation state of DCs to improve clinical outcomes.

### Form of Antigen

Another variable amongst the different DC vaccination strategy is the type and form of antigen loaded onto DC.

#### Peptide or Protein

The most common source of antigen is protein. Early DC vaccines use short peptide sequences unique to tumor associated antigens that are known to bind to specific HLA subtypes, mainly HLA-A2. Short peptides are easy to make and are quickly presented on MHC class I by DC when added to culture media. However, they have several disadvantages. They must be suitable for that patient's HLA subtype or else, as they will not be presented, and immune responses will be limited (18). Whilst several vaccines have been trialed selecting patients of HLA-A2 subtype this excludes at least half of eligible patients and represents a higher percentage of the Caucasian population than other ethnics backgrounds (70). Additionally, short peptides that target a CD8^+^ T cell response won't harness CD4^+^ T cell help limiting T cell expansion, cytotoxicity, and memory (71). MHC Class II molecules are more variable than MHC class I and thus designing short peptides to target them as well as MHC class I to cover large proportions of the populations becomes complicated and difficult to standardize.

The limitations of peptide loading can be overcome by administering whole protein for DC to uptake and process. Recombinant protein is easy to obtain and can be added directly to culture media. The advantage of administering whole protein is that after DC processing, multiple peptides are available that bind both MHC class I and II and multiple HLA types. The disadvantages are that these proteins may not cover the potentially more immunogenic mutations found in the tumor and reagents to monitor peptide specific responses may not be available. Also, by focusing on one to four proteins, this leaves open the possibility of immune escape as tumors down regulate expression of these proteins. In the case of PCa, most proteins used in clinical trial including PSA, PSMA, and PAP are overexpressed self-antigen and thus have issue with self-tolerance.

#### Tumor Cells

Cell lysate has the benefit of presenting a multitude of tumor protein both known and unknown, as well as mutated protein found in the tumor. These mutated proteins give rise to neo-antigens that overcome the problem of self-tolerance and thus are more immunogenic. A common way to produce cell lysate is to freeze/thaw cells for several cycles producing necrotic cell death. This process leaves cell membrane fragments, RNA and DNA in the lysate which provide danger signals promoting DC maturation (72). Once produced cell lysate is added to culture media at ratios of 5:1 (45) and up to 1:1. This requires access to a large amount of tumor material, which, particularly in the setting of CRPCa, is difficult. This has led to the use of allogeneic cell lines as surrogate tumor tissue in four clinical trials in PCa (25, 35, 45, 50) (Table 1). Two of these trials combine treatment with metronomic cyclophosphamide for 7 days prior to DC vaccination (45, 50). These trials show that the use of tumor lysate is safe and produces a tumor-specific immunological response as well as increasing PSADT (36, 50).

Allogeneic apoptotic tumor cells (36) have similar capacity as cell lysate to mature DC and prime T cells to produce an antigen specific immune response (73). Apoptotic tumor cells are effectively phagocytosed by immature DC (74–76) and their tumor antigens are preferentially cross-presented to CD8^+^ T cells. A melanoma mouse models suggest that apoptotic tumor cells induces more IL-12 secretion by DC than cell lysate (73). In patients with CLL *in vivo* studies support this finding show that apoptotic tumor cell loaded MoDC produce better T cell proliferation, higher frequency of IFNγ producing T cells *via* ELISPOT and by PCR less mRNA for the Th2 cytokines IL-4 and IL-10 than cell lysate and mRNA pulsed MoDC (77).

Other forms of presenting tumor antigen to DC include producing hybrids of DC and tumor cells fused using polyethylene glycol (PEG). These made *in vitro* using PCa cell lines ONYCAP23, P4E6, and LNCaP and MoDC, can produce a tumor cell-specific immune response (78). Conceptually, by fusing the cells, endogenous tumor antigens have better access to MHC class I molecules. Several early phase I/II clinical trials in melanoma, glioma, renal cell carcinoma, breast cancer demonstrate that this is feasible, safe, and produces clinical responses (79).

Exomes provide an acellular source of tumor antigen. Exomes are nano-sized particles originating from multivesicular bodies. They can be isolated from the blood and urine of PCa patients (80) providing a source of current antigenic material that is often difficult to obtain in mCRPCa and facilitating a mechanism for a personalized vaccine. Exomes have long been known to have immunosuppressive properties (81), suppressing T cell and NK cell function in the tumor microenvironment. In direct contrast to this, when exosomes activate DC which activate tumor specific T cells as effectively as cell lysate (81, 82). This creates a promising pathway for future autologous prostate cancer tumor loaded DC vaccines.

#### Messenger RNA

Finally, mRNA provides another source of antigen (74), which DC can take up and translate into protein for presentation on MHC class I. mRNA has the advantage that it can be prepared in sufficient quantity from a small tumor sample and thus it also allows for the ability to produce personalized vaccines. There are four ways of administering mRNA to the DC (a) passive, (b) liposome mediated, (c) electroporation, and (d) viral vector mediated. By far the most common way is electroporation. This has been done in a phase II trial in PCa that compares mRNA loaded MoDC in combination with docetaxel to docetaxel alone (46) (Table 1). Whilst it was deemed to be safe with the only toxicity identified as related to vaccine local reactions and rash, there was a much higher discontinuation of treatment in the vaccine arm-−57 vs. 21%. The vaccine arm required much more frequent visits, however, as reasons for discontinuation where not reported, additionally toxicity cannot be excluded.

### Route of Administration

MoDC vaccines have been administered in multiple different routes including intravenous (I.V.), intranodal (I.N.), intralymphatic (I.L), intradermal (I.D.), and subcutaneous (S.C). In a meta-analysis that pooled individual data from 84 patients, routes that allow migration to local lymph nodes i.e., I.D./I.L./I.N./S.C lead to better clinical response compared to the I.V. route (OR 3.2, 95% CI 1.1–9.0) (56). Fong et al. (41) showed similar findings using density enriched DC with a better cytokine profile seen with I.D. and I.L. route compared to I.V. with a trend to more transfusion reactions in the I.V. group.

Despite the variability in preparation as a whole these trials (Table 1) show that MoDC vaccinations in PCa are safe, produce a cellular immune response and a clinical response with a fall in PSA seen in up 27% (9/33) (18, 50, 83). However, it is important to note that an immunological response does not necessarily correlate with outcome (45, 46, 50) and often peripheral immune responses when detected are not sustained (46). Thus, the outcomes measured may not be clinically significant. Surrogate endpoints of reduction in PSA and difference in PFS may also not correlated with OS, as seen with Sipuleucel-T (5). Thus, despite a multitude of early trials we really need a Phase III trial of MoDC that looks at OS to determine clinical significance. The results of NCT02111577, a double blinded Phase III trial of MoDC loaded with apoptotic LNCaP cells added to standard chemotherapy for men with mCRPCa which has completed recruitment with 1,182 patients, should provide us with some clearer answers.

However, even without the results of this trial there are a number of reasons why MoDC preparations may not be the optimal approach. Monocytes are known to be dysfunctional in advanced cancer including in PCa. Most preclinical information on MoDC has been collected using healthy donor PBMC. However, when we compare MoDC prepared from healthy donors to those from patients with advanced cancer, patient MoDC are less efficient at phagocytosis, produce less IL-12 and express lower levels of the activation marker CD80 (84). In study of 24 patients with localized PCa, MoDC failed to upregulate CD80, CD83, and CCR7 after maturation with human cytokine cocktail, although for most patients, but not all, this was restored after surgery (85). In contrast two studies of only five patients each did show that MoDC from PCa patients were as good as healthy donors (32, 86).

The biggest issue with MoDC is that even from healthy donor PBMC, they do not perform as well as blood-derived DC; they do not stimulate T cells as well (54, 87), migrate as well (88) or have as much clinical efficacy (56). Thus, despite ease of production, MoDC vaccinations are unlikely to improve on the effectiveness of Sipuleucel-T.

### Blood Derived DC Vaccines

Advances in efficiency of isolation protocols allow the use of blood DC as an alternative to MoDC or DC enriched preparations (Figure 1). Prue et al. (43) in a phase I trial showed that it was feasible to isolate CD1c^+^ DC from CRPCa patients *via* magnetic separation and vaccination, was well-tolerated with fever and pain the most common toxicity (43). More recently, in a phase II RCT Westdorp compared the efficacy of matured myeloid (m)DC vs. plasmacytoid (p)DC vs. combination of mDC and pDC (44). Again, this showed that blood derived DC were safe and induced an immune response, with a trend to a better response with mDC alone. These technical advances in isolating DC as a pure population and, as demonstrated by Westdorp et al. (44) isolating specific DC subsets and utilize the underlying specialization of human DC to take up antigen allows us to direct the immune response in a particular direction.

#### Targeting DC Subsets

Blood DC can initially be divided into two main populations: pDC and mDC. Human pDC are identified by their surface expression of CD304^+^. They are characterized by their ability to produce large amounts of Type 1 interferon in response to foreign nucleic acids i.e., in response to viral infections (89). In humans, they orchestrate antigen specific CD4^+^ T cell responses as well as cross present antigen to create CD8^+^ T cell responses (90, 91). mDC, divided based on phenotype and function into five subsets, the main being cDC1 and cDC2 (92). cDC1, characterized by CD141 expression, have the ability to cross present exogenous antigen to prime CD8^+^ T cell response, direct a type 1 helper T cell responses and through the production of IL-12, and direct an NK response (93). cDC2, are characterized by CD1c^+^ expression, have a more diverse function and are able to simulate Th1, Th2, Th17, and CD8^+^ T cell responses (93). As suggested by Westdorp et al. (44) the mix of DC we use for a vaccine will affect efficacy (44). Whether we use a mixed preparation of mononuclear cells, DC, T cells, B cells, and NK cells such as in Sipuleucel-T (94), MoDC or a pure DC subset will change the direction of the T cell response. Traditionally we have looked at mDC particularly cDC1, as key to orchestrating a cytotoxic immune response. They are most adept at priming CD8^+^ T cells because they have adapted their intracellular machinery to be extremely efficient at cross presentation of antigen (95). Whilst they have been the focus of much vaccine development, as we learn more about the need for T helper support to create effective CD8^+^ T cell response (96, 97) an approach that utilizes both cDC1 to activate CD8^+^ T cells and cDC2 to activate CD4^+^ T cells would give a more robust anti-tumor immune response (98). A novel way of targeting these naturally occurring DC is to target DC *in situ*. Emerging technologies such as antibody-antigen conjugates and virus co-delivery systems not only provide a DC therapy that improve delivery they also improve efficacy.

## *In Situ* DC Targeting

### Antibody Directed Antigen-Uptake

One way to target DC is to couple antigen to antibodies that bind endocytic cell surface molecules unique to DC. Preclinical data in mouse models show that delivering antigen in this way increases the efficiency of antigen presentation. Coupling OVA to the rat anti-mouse DEC-205 antibody (clone: NLDC-145) lead to a >100-fold increase in efficiency of DC antigen presented to mouse CD4^+^ and CD8^+^ T cells (99). Thus, targeting antigen directly to DC with antibody increases antigen presentation and in both *in vivo* mouse models and *in vitro* human models this leads to improved T cell response (100–102). However, in the absence of a maturation signal to the DC or indeed as a consequence of the function of the molecule targeted, this T cell response did not persist and in fact peripheral tolerance was induced (99). In contrast, in the presence of adjuvant such as anti-CD40 a strong memory response is formed after injection with OVA conjugated DEC-205, with CTL responses detectable up to 90 days after a single immunization (100). This need for a second “danger” signal to direct the immune system to form an inflammatory rather than tolerogenic response to the targeted antigen is not unique to DEC-205 antibodies but common to many surface antibody targets studied to date (99, 100, 103, 104). However, the selection of adjuvant in a clinical setting will need careful consideration to minimize side effects.

Despite the need for adjuvant, the safety and ease of delivery of *in vivo* DC targeting has been demonstrated in a phase I clinical trial of CDX-1401, a fully human anti-DEC-205 (CD205) mAb (3G9) genetically fused to the full-length NY-ESO-1 protein. The vaccine was used in combination with resiquimod (TLR7/8 agonists) and poly-(I:C) as adjuvants. It was well-tolerated in the 45 patients who entered the study and, induced a cellular immune response in 56% and humoral immune response in 79% of cases. Thirteen patients developed stable disease and 2 a partial response (105). This demonstrates that using antibody to target antigen to DC is safe and feasible and can induce an immune response in humans.

While safety has been demonstrated, reports on trials in ovarian cancer and acute myeloid leukemia (AML) are awaited. There remains the question of choice of molecule to target as targeting DEC-205 which naturally trends toward tolerance may be superceeded. Clec9a (CD370) is another endocytic surface marker with a much narrower expression profile. Whilst DEC-205 is highly expressed on cDC1 it is also expressed on monocytes, B lymphocytes and low levels on T cells and NK cells. In contrast, Clec-9A expression is limited to cDC1, which is the DC subset known for their ability to cross present antigen and elicit a CD8^+^ cytotoxic T cell response, ideal for a tumor vaccine. The other interesting ability of Clec9a is its ability to drive a memory immune response without adjuvant, however, in mice tumor models, adjuvant is still required (106). Whilst this looks like a promising target, it has been demonstrated that the cDC1 population, which is targeted by Clec9a is reduced in PCa (107) and is less responsive to activation with poly (I:C). This suggests underlying functional impairment and testing these treatments in a PCa tumor model is awaited.

Antibody-directed antigen uptake demonstrates that DC can be loaded “*in vivo*” (105), is safe and produces an immune response. However, antibodies are limited by the amount of antigen that they can deliver through coupling protein to antibody before the latter's ability to bind and be endocytosed is impaired. This has led to the development of co-delivery systems.

### Co-delivery Systems

Co-delivery systems have two advantages, they allow the co-administration of adjuvant with antigen and can deliver multiple antigens. Some co-delivery systems are easy to adapt to different antigen make ups thus allowing personalized vaccine with “neoantigens' matched to each patient. There are two main vehicles studied: modified viral vectors and nanoparticles. Viral vectors include the filamentous bacteriophage antigen display system and modified adenovirus. The filamentous bacteriophage system is based on a non-pathogenic prokaryotic virus which can be engineered to express exogenous peptides as fusions to viral capsid proteins (108, 109). The bacteriophage is the adjuvant and in a mouse model it has been manipulated to express both mouse DEC-205 and OVA. In this system it produces an enhanced T cell response compared to injection with OVA: DEC-205 antibody conjugate (108). Similarly, a model where attenuated adenovirus was manipulated to express OVA and anti-mouse DEC-205 (110), produced a memory CD8 T cell response. Whilst this shows promise in pre-clinical models, translation to humans is yet to come.

### Nanoparticles

Nanoparticles perhaps are closer to translation, in particular Poly(DL-lactide-co-glycolide (PLGA), a biodegradable slow-release polymer that is FDA approved to encapsulate drugs, can be adapted to encapsulate antigen and adjuvant (111). Due to their size nanoparticles readily taken up by DC (112) and *in vitro* studies show human DC take up peptide more efficiently if it is delivered inside a PLGA nanoparticle rather than soluble form (113). Nanoparticles not only direct peptide to the DC but also protect peptide from degradation, thus increasing the length of time to which DC are exposed to peptide. PLGA delivery of peptide induced T cells with a much greater CTL response than peptide loaded DC both *in vitro* (113) and *in vivo* (113, 114). Nanoparticle delivery has been tested in a mouse models of prostate cancer with the mouse prostate tumor antigen, six-transmembrane epithelial antigen of the prostate (mSTEAP). In this model, a single dose of mSTEAP on PLGA nanoparticles was compared to mSTEAP peptide plus adjuvant. The nanoparticle bound mSTEAP reduced both growth of TRAMP-C2 tumor cells in C57BL/6 mice and increased OS of the mice compared to peptide combined with adjuvant (114). Thus, in a PCa model, nanoparticles were more effective than a peptide vaccine. It is important to note though that comparison to DC vaccination strategies, antibody directed antigen uptake or other novel vaccination strategies remains to be assessed.

Nanoparticles have been used as a co-delivery system for antibody directed antigen uptake. Nanoparticles coated in anti-DEC-205, anti-CD40, and anti-CD11c antibodies to deliver antigen and adjuvant direct to DC all lead to increased CD8 and CD4 T cell proliferation and cytotoxicity *in vitro* and *in vivo* above non-targeted nanoparticles. CD40 targeted nanoparticles improved antigen specific T cell proliferation in the draining LN above other target receptors, and also cytotoxicity against target cells (115). In a mouse tumor model, CD40 nanoparticles containing OVA improved OS of B16-OVA inoculated mice compared to isotype control (116).

Whilst these emerging technology show promise in improving deliverability and efficacy of a DC based vaccine, they are yet to be translated into clinical trials in prostate cancer.

### Overcoming Tumor Escape

If we are to successfully translate *in situ* targeting of DC, clinical benefit will not occur without understanding what drives the immunosuppressive microenvironment of PCa.

#### Improving Antigen Processing Within Tumor Cells

PCa evades detection of the immune system by failing to display tumor peptide in MHC class I complexes on their cell surface. This is crucial to consider in the setting of DC vaccine as cytotoxic T cells primed by a DC vaccine will not be able to kill tumor cells without the presence of MHC Class I complex on tumor cells. In primary castrate sensitive prostate cancer (CSPCa) MHC class I was downregulated in 74% (311/419) and β2M 25% (117). In another study of 58 primary CSPC, defects in MHC class I were less common with loss of staining only in 5% of cases but heterogenous staining in 62% (117). This study also looked at the components of the antigen processing machinery within the tumor cells and demonstrated that loss or downregulation was frequent (118). Thus, treatment strategies that increase MHC Class I expression on tumor cells are candidates for combination therapies that may improve efficacy of DC vaccines. Histone deacetylase inhibitors have been assessed to reverse histone acetylation of the TAP1 promotor and, Trichostatin A, has been shown to upregulated MHC-class 1 and β2-microglobulin in LNCaP cells. Traditional anti-PCa therapies such as docetaxel and radiation also increase all components of antigen-processing machinery in the PC cell line, LNCaP (119, 120) and therefore are beneficial combination strategies for DC vaccines. A phase II trial that combined MoDC vaccine with docetaxel showed a trend toward improvement in disease specific survival (DSS) (46), and results of the first phase III trial, NCT NCT02111577 that combines docetaxel and DC vaccine therapy are eagerly awaited.

#### Improving T Cell Function

A robust T cell response is essential for any effective DC vaccine. Thus, it is essential to understand any underlying dysfunction of the T cell repertoire in PCa. We know there are a paucity of T cells (121) in PCa and those present are less proliferative (122), more immunosuppressive (123) with a high proportion of T-regs (122, 123). Data from the NCT00715014 trial of neoadjuvant Sipuleucel-T shows that DC vaccination does lead to increased recruitment of T cells including CD4^+^, CD8^+^, and T-regs into the tumor (12). Comparing pre vaccination biopsies to post vaccination resection specimens, T cells had increased TCR sequence diversity in the resected prostate suggesting that Sipuleucel-T recruits T cells to the prostate (124) rather than reactivating those already *in situ*. Gene expression profiling showed an increase in Th1 associated genes and upregulation of immune checkpoint inhibitors including CTLA-4 and TIGIT (125). This raises the question of how long does the immune response last and whether combining with check point inhibitors will improve outcomes.

While monotherapy with both ipilimumab (anti-CTLA4) and PD-1 inhibitors have proved disappointing (2, 3, 126), recent long term follow of ipilimumab shows that despite low response rates those that do respond have enduring responses (127). The key will be to improve response rates and early data suggests that adding DC vaccination to immunotherapy may do just that. In a small study of nine men with mCRPCa treated with Sipuleucel-T and escalating doses of ipilimumab showed that IgG and IgM levels against PA2024 and PAP increased significantly after ipilipumab (49). A subsequent trial to look at immediate vs. delayed CTLA4 blockade (NCT01804465) has recruited and is in the follow up stage. PD-1 inhibitors have less severe immune toxicity than anti-CTLA4 antibodies, and thus are a more tolerable combination strategy. Pembrolizumab has been used in combination with a DNA vaccine in PCa and it was found that concurrent rather than sequential treatment improved PSA response (128). We look to the results of NCT03024216 to determine whether atezolizumab (anti-PD-L1) improves the efficacy of Sipuleucel-T.

Another strategy is to focus on depleting T-regs. In mouse models of PCa low dose cyclophosphamide caused transient depletion of T-regs and increased DC maturation markers and augmented anti-tumor immune response (129). In humans, metronomic oral cyclophosphamide was used in combination with a MoDC vaccine (50), and also prior to MoDC vaccine used in combination with docetaxel chemotherapy (45). In both instances it was well-tolerated. Another mechanism to reduce T-regs is to use IDO inhibitors to block the production of IDO-expressing DCs that drive T cells to T-regs and activate existing T regs. Indoximod, an IDO inhibitor administered after Sipuelucel-T therapy was found to be well-tolerated and improved PFS from 4.1 to 10.3 (*p* = 0.011) (53).

#### Over-coming Myeloid Derived Suppressor Cells

Myeloid cells play a large role in creating the tumor microenvironment of PCa. The presence of M2 macrophages in the tumor microenvironment is an indicator of poor prognosis (130–133). PCa cells recruit monocytes and polarize them to an M2 macrophage phenotype which then helps increase PCa cells migratory capacity, proliferation, survival and invasion (130, 134, 135) creating a symbiotic relationship. Interestingly, a reduction in MDSC predicts response (46) to mRNA loaded MoDC vaccination and tumor cell vaccine in combination with ipilimumab (136). In mice models of lung cancer MDSC reduce the activity of NK cells and T cells, thus, they will dampen any immune response developed by a DC vaccine. Novel combination strategies that further reduce MDSC may improve vaccination responses. Interestingly in a breast cancer tumor model docetaxel repolarized MDSC toward an M1-like phenotype further supporting the use of docetaxel as a combination for vaccination (137).

#### Timing and Interactions of Other Therapies

It has long been proposed that the best time to treat with a DC vaccine is when tumor burden is low either at diagnosis or remission. This hypothesis is supported by trials that low burden of disease predicts for good response (138). Another issue is the effect of treatment on the immune system's ability to create an immune response. In the instance of PCa, androgen deprivation therapy (ADT) is given throughout the entire treatment course. ADT enhances T cell responses. In a mouse model, after androgen withdrawal the biggest difference in CD4^+^ T cells was in IFNγ signaling pathway and CD4 T helper differentiation (139). In patients in CSPCa this was also the case (140). However, we also know that these responses diminish with time, perhaps due to a disproportionate increase in T-regs (141). In a mouse model depleting T-regs with a CTLA-4 depleting antibody significantly improved OS when combined with ADT (142). The phase II STAND study assesses this in patients and showed that better immune responses were stimulated when a DC vaccine was given before initiation of ADT rather than after (48). Thus, the best timing for a DC vaccine maybe at biochemical recurrence when tumor burden is low and ADT has not been given.

## Conclusion

DC vaccination strategies have been shown to be safe and improve OS. Yet they are still rarely used in clinical practice. Our understanding of antigen loading DC, antigen presentation, induction of T cell responses, extrinsic driving of cytotoxic responses provides multiple opportunities to improve vaccine strategy design. Here we show that emerging technologies present options for targeting DC *in situ* thus improving deliverability. Secondly, novel combination strategies prove promising to help improve on duration of T cell response. That DC vaccines reach their potential in stimulating effective clinical responses relies on assessing what we have learned, how we adapt trials and looking for long term, durable (or sustainable) outcomes.

## Author Contributions

SS: writing and figures. XJ, LH and GC: reviewed. SS and GC: concept. All authors contributed to the article and approved the submitted version.

## Conflict of Interest

The authors declare that the research was conducted in the absence of any commercial or financial relationships that could be construed as a potential conflict of interest.
